# Hyperglycosylation as an Indicator of Aging in the Bone Metabolome of *Oryzias latipes*

**DOI:** 10.3390/metabo14100525

**Published:** 2024-09-27

**Authors:** Remi O. Labeille, Justin Elliott, Hussain Abdulla, Frauke Seemann

**Affiliations:** 1 Department of Physical and Environmental Sciences, Texas A&M University-Corpus Christi, Corpus Christi, TX 78412, USA; rlabeille@tamucc.edu (R.O.L.); jelliott11@islander.tamucc.edu (J.E.); hussain.abdulla@tamucc.edu (H.A.); 2 Department of Life Sciences, Texas A&M University-Corpus Christi, Corpus Christi, TX 78412, USA

**Keywords:** medaka, glycosylation, bone senescence, hexosamine biosynthetic pathway, IC-MS/MS

## Abstract

Chronological aging of bone tissues is a multi-faceted process that involves a complex interplay of cellular, biochemical, and molecular mechanisms. Metabolites play a crucial role for bone homeostasis, and a changed metabolome is indicative for bone aging, although bone metabolomics are currently understudied. The vertebral bone metabolome of the model fish Japanese medaka (*Oryzias latipes*) was employed to identify sex-specific markers of bone aging. 265 and 213 metabolites were differently expressed in 8-month-old vs. 3-month-old female and male fish, respectively. The untargeted metabolomics pathway enrichment analysis indicated a sex-independent increased hyperglycosylation in 8-month-old individuals. The upregulated glycosylation pathways included glycosphingolipids, glycosylphosphatidylinositol anchors, O-glycans, and N-glycans. UDP-sugars and sialic acid were found to be major drivers in regulating glycosylation pathways and metabolic flux. The data indicate a disruption of protein processing at the endoplasmic reticulum and changes in O-glycan biosynthesis. Dysregulation of glycosylation, particularly through the hexosamine biosynthetic pathway, may contribute to bone aging and age-related bone loss. The results warrant further investigation into the functional involvement of increased glycosylation in bone aging. The potential of glycan-based biomarkers as early warning systems for bone aging should be explored and would aid in an advanced understanding of the progression of bone diseases such as osteoporosis.

## 1. Introduction

Skeletal tissue homeostasis is maintained through a delicate balance of osteoblast and osteoclast activity, and disruptions in this balance can lead to various skeletal changes. Age-related bone loss, a hallmark of osteoporosis, has been extensively studied in the context of dysregulated osteoblast and osteoclast function [1,2]. However, the precise molecular mechanisms underpinning skeletal aging remain incompletely understood. Teleost fish, such as the Japanese medaka (*Oryzias latipes*), have emerged as powerful models for investigating skeletal biology due to their evolutionary conserved spinal structures and rapid life cycles [1]. Japanese medaka and mammals differ mainly in the absence of osteocytes in their skeletal tissues. However, the molecular machinery that regulates bone adaptation to mechanical loads is conserved [3,4]. The Japanese medaka fish has a well-established history as a vertebrate biomedical model with demonstrated translatability to human disease research [2,5,6]. The strengths of medaka for this research include the availability of multiple transgenic lines [7,8,9,10]; the transparency of eggs and larvae, which enables in vivo assessment of bone development and real-time monitoring of reporter gene expression [10], established protocols for various omics applications [11,12], and the availability of a high-quality sequenced genome and epigenome [12,13,14,15]. The similarity of mammalian and medaka molecular markers and pathways during bone formation, bone cell differentiation, and bone metabolism [8,16] allows for the translation of research findings on genetic and epigenetic responses to other vertebrates, including humans.

The emergence of medaka as a model organism for skeletal research has prompted the need to establish a baseline bone tissue metabolome across sex and age. Bone aging is associated with bone loss, which has been attributed to both systemic chronic inflammation and osteoblast/osteoclast dysfunction [17]. Aging generates a senescence-associated secretory phenotype (SASPs) due to an increased presence of senescent cells, including T-cells, B-cells, osteoblasts, and osteoclasts [17,18,19]. Chronological aging of bone tissues is a multi-faceted process that involves complex interplays of cellular, biochemical, and molecular mechanisms that remain inadequately described. Proinflammatory cytokines such as Interleukin-1, Interleukin-6, and Tumor Necrosis Factor have been shown to hinder bone formation and fracture healing, making them potential candidates for T-cell SASPs in bone tissue [17]. In addition, the signaling pathways of bone cells, such as receptor activator of nuclear factor kappa-Β ligand (RANKL) and Osteopontin N-glycosylation (OPN) for osteoclasts, as well as leptin transforming growth factor beta 1 in osteoblasts, are dysfunctional due to aging [20,21,22]. However, the mechanisms that initiate and promote cell senescence in bone tissue are not well understood.

The age of 8 months in *O. latipes* has been reported as the starting point of estrogen depletion and the onset of bone mineralization decline in female fish [1,23]. In addition, sex differences in immune gene expression were reported during this life span [24].

Altered metabolism is known to be directly implicated in tissue inflammation and cellular senescence, but to what extent is unclear [25]. Research on bone tissue aging suggests a potential role for glycosylation, as indicated by in vivo and in vitro analysis of the transcriptome and proteome [2,20,26,27]. Fine-tuning of glycosylation metabolism can directly affect cell-cell recognition and signaling pathways by altering proteoglycans and liposaccharides [21,28]. The modulation of glycosylation pathways has been shown to affect osteoblast and osteoclast development and function, suggesting a potential role in skeletal aging but it has not been thoroughly examined. Deletion of β1,4-N-acetylgalactosaminyltrasferase in mice resulted in reduced N-acetylgalactosamine synthesis, leading to decreased bone formation and a reduction in osteoblast count [2]. O-Linked β-N-acetylglucosamine (O-GlcNAc) plays a crucial role in regulating osteoblast differentiation by modifying key proteins such as Runt-related transcription factor 2 (Runx2), TGF-beta activated kinase 1 (TAB2), and CREB-binding protein (CBP) to enhance the signaling pathways [2]. OPN, a major bone matrix protein, requires glycosylation for its mineralization function, and changes in its glycosylation patterns have been associated with age-related bone loss [22]. Additionally, OPN N-glycosylation modulates the expression of osteoclast- and osteoblast-associated factors through the Nuclear Factor kappa-light-chain-enhancer signaling pathway (NF-κB) [22]. N-glycosylation of OPN promotes nuclear translocation of NF-κB in both osteoclasts and osteoblasts [22]. The coordination of bone transcriptional networks is linked to the use of nutrients and metabolites for decorating both proteins and signaling molecules. O-GlcNAc acylation is highly sensitive to nutrients as it relies on Uridine-diphosphate-N-acetylglucosamine (UDP-GlcNAc), the final product of the Hexosamine Biosynthetic Pathway (HBP) [21]. Aging bone tissue has also been shown to cause endoplasmic reticulum (ER) stress, which is hypothesized to alter post translational glycosylation, but this has not yet been investigated. Collectively, the glycosylation–metabolite axis plays a crucial role in bone remodeling.

These findings highlight the pivotal role of glycosylation and associated metabolites in regulating bone cell functions. Thus, a more comprehensive investigation focusing on the differential glycosylation profiles across sex and age is warranted. Utilizing an untargeted metabolomics approach, the global metabolomic landscape of medaka skeletal tissue was characterized, with a particular focus on elucidating age-related and sex-specific changes in glycosylation pathways. Our limited knowledge regarding glycosylation states in tissue lies in instrumental limitations. Recent advancements in untargeted ion chromatography (IC) coupled with the Orbitrap Fusion Tribrid Mass Spectrometer (OT-FTMS) have enabled comprehensive profiling of complex glycans, revealing their integral role in cellular signaling and tissue homeostasis [29]. OT-FTMS enables simultaneous identification and quantification by utilizing two mass analyzers operating in parallel, delivering unparalleled speed, selectivity, accuracy, sensitivity, and high reproducibility. Additionally, our newly developed on-the-fly locking technique ensures high mass accuracy for every scan by introducing internal labeled standards post-column [29]. This method consistently achieves mass errors below 1.0 ppm. Coupling IC with OT-FTMS significantly enhances sensitivity for detecting negatively charged metabolites compared to the traditional coupling of LC with Orbitrap MS. Here, we leverage the unique advantages of the medaka model to provide novel insights into the metabolic underpinnings of skeletal aging and sex differences, with implications for improved diagnostic and therapeutic interventions.

## 2. Materials and Methods

### 2.1. Medaka Bone Cell Isolation

**Figure 1:**
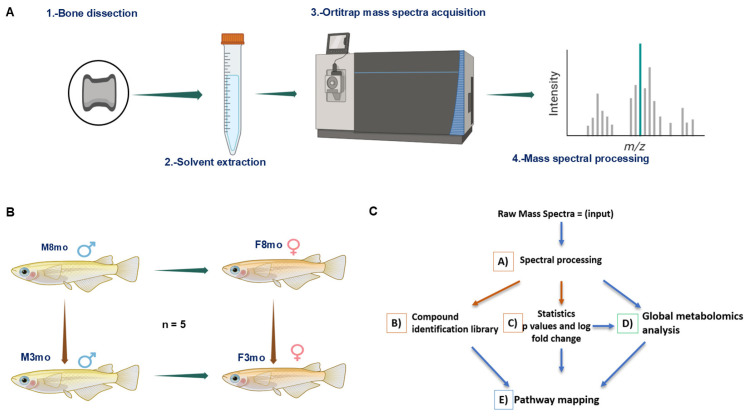
Schematic of metabolite acquisition (**A**) using orbitrap MS in negative mode, multiple pairwise statistical comparisons between sex and age (**B**), and bioinformatics pipeline (**C**).

Male and female medaka were raised until 3 months of age and 8 months of age following the standard protocol from [30]. All animal research was completed under the TAMU-CC IACUC approval (TAMU-CC-IACUC-2023-0003; TAMU-CC-IACUC-2023-0002). Vertebrate bone was dissected from male and female fish (n = 5 per sex and age; Figure 1). The bone cells were dissociated using a modified protocol from [31]. Specifically, the vertebrae were dissociated using 500 μL of dissociation mix (0.55% collagenase, 1% trypsin/EDTA, and 98.5% PBS) in a 2.0 mL microfuge tube and incubated for 30–40 min with shaking on a thermomixer at 30 °C. Every 10 min, the tubes were removed from the thermomixer and the solution was mixed. 55 μL of fetal bovine serum was added to stop the dissociation reaction, followed by an additional 3 min of shaking on the thermomixer at 30 °C. The 2 mL microtubes were then placed immediately on ice for 1–3 min until all non-digested debris accumulated at the bottom. Next, 400 μL of the supernatant was transferred to a new 2.0 mL tube. Then, 500 μL of PBS was added to the first tube containing the bone matrix and chilled on ice for 1–3 min. Next, 400 μL of the supernatant from the first tube was combined with the 400 μL in the second microtube. The supernatant was centrifuged at 4 °C for 5 min at 600× *g*. The supernatant was removed, and the pellet was resuspended in 1 mL of PBS. The resuspended pellet solution was then filtered through a cell strainer (70 µm), and the filtered solution containing the cells was kept on ice. Finally, cell viability and cell numbers were assessed with 100 ng/mL of DAPI. Cell viability was above 90% for all samples. Cells were adjusted to a density of 10^6^ cells/mL. Cell pellets were snap-frozen in liquid nitrogen and stored at −80 °C.

### 2.2. Metabolite Extraction and Orbitrap Mass Spectrometry Analysis

The bone cells from the skeletal tissue were homogenized using methanol digestion. The polar metabolites were extracted via sonication and centrifuged, and the supernatant was used for the IC-MS analysis [32]. Spectral acquisition was accomplished on a Dionex IC5000+ system coupled to an Orbitrap Fusion Tribrid mass spectrometer. Metabolite separation was achieved with an AS11-HC column (200 nm, 4 μm × 2 mm × 250 mm) and AG11-HC guard column (4 μm, 2 mm × 50 mm) using potassium hydroxide [KOH] from an automated eluent generator. A 30-min gradient went as follows: 1 mM 0–5 min; 1 mM to 4 mM, 5–20 min; and 4 mM to 60 mM, 20–30 min at a flow rate of 0.4 mL/min. To improve mass accuracy and enhance ionization, a T joint intersection post-separation infused a solution of C13 Hippuric acid for on-the-fly lock mass calibration at a 0.2 mL/min flow rate. Polar metabolites underwent heated electrospray ionization (ESI) with parameters as follows: spray voltage, −3 kV; sheath gas, 50; auxiliary gas, 20; sweep gas, 2; vaporization temperature, 300 °C; ion transfer tube temperature, 350 °C; and radio frequency (RF) lens level, 40. Data-dependent acquisition (DDA) was used to acquire full scan MS1 spectra at a resolution of 120,000 (FWHM at 200 *m*/*z*) with a scan range of 85–700 *m*/*z*, automatic gain control (AGC) target 3 × 10^3^, and maximum injection time of 50 ms. MS2 was subject to an intensity filter of 3 × 10^3^ and dynamic exclusion with 10 ppm mass tolerance for 30 s. Fragmentation occurred via collision-induced dissociation (CID) or higher-energy collisional dissociation (HCD) with assisted fragmentation energy [32].

Compound Discoverer software 2.0 (Thermo Fisher, Waltham, MA, USA) was used to identify the mass features and group them into individual compounds. De novo structural elucidation was performed on compounds with MS2 and annotated by spectral library matching, including mzCloud, HMDB, METLIN, MassBank, NIST, in-house polar metabolite libraries, and in-silico fragmentation via FiSH (fragment ion search hierarchy) scoring. Molecular formulae were determined by a combination of OT-FTMS accurate mass and isotope pattern matching to confirm the charge state and likely formula. Tentatively identified and unidentified metabolites were exported for bioinformatic analysis.

Pathway enrichment analysis was performed using the mummichog2 algorithm in MetaboAnalystR 3.0 [33]. The peak list containing the mass to charge (*m*/*z*) value was used without the relative peak intensities. The zebrafish (*Danio rerio*) metabolome was used as the reference metabolome (GitHub for code). Pathway enrichment data (GitHub for data) along with compound identifications through means of empirical compound matches were obtained. Pathways with statistically significant *p* (gamma) < 0.05 and at least three identified empirical metabolites were subjected to metabolic pathway mapping. Mapped metabolites included peak lists from both MS/MS Compound Discoverer 2.0 and MetaboanalystR 3.0, with a *p*-value < 0.05 and cut-off values of −1 > log2 fold change and log2 fold change > 1. Metabolic pathway mapping was accomplished through the SBGNview library [34] (see GitHub for code).

During the preparation of this work, the author(s) used Jenni AI (https://jenni.ai/; accessed on 22 September 2024) to refine the sentence structure of the initial draft of the introduction and discussion. The MS has subsequently undergone multiple rounds of revision through the co-authors, reformulating the AI suggestions.

## 3. Results

### 3.1. Compound Identification

**Figure 2:**
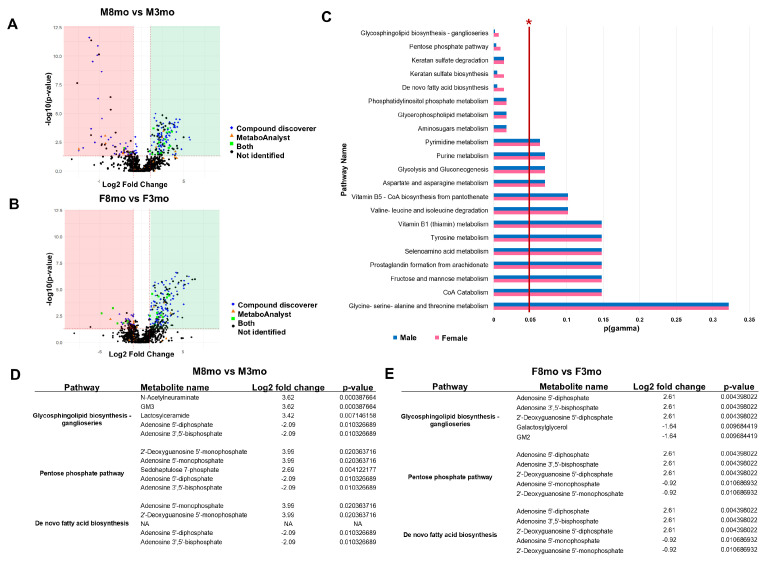
Metabolomics data comparing age of both sexes through volcano plots, pathway enrichment analysis and the top 10 statistically significant identified metabolites. (**A**) Age comparison of M8mo vs. M3mo (blue: compound discoverer matched, orange: MetaboAnalyst empirical compound matched, green: compound discoverer and MetaboAnalyst empirical compound matched). (**B**) Age comparison of F8mo vs. F3mo (blue: compound discoverer matched, orange: MetaboAnalyst empirical compound matched, green: compound discoverer and MetaboAnalyst empirical compound matched. (**C**) Statistically significant enriched metabolic pathways with at least three statistically significant metabolites (pink = F8mo vs. F3mo, blue = M8mo vs. M3mo) and a red vertical asymptote at *p* = 0.05 indicated as well by a red star. (**D**) Table of the metabolites with the highest and lowest log2 fold change values in the M8mo vs. M3mo comparison, including Log2 fold change, *p*-value, and the respective metabolic pathways. (**E**) Table of the metabolites with the highest and lowest log2 fold change values in the F8mo vs. F3mo comparison, including Log2 fold change, *p*-value, and the respective metabolic pathways.

**Figure 3:**
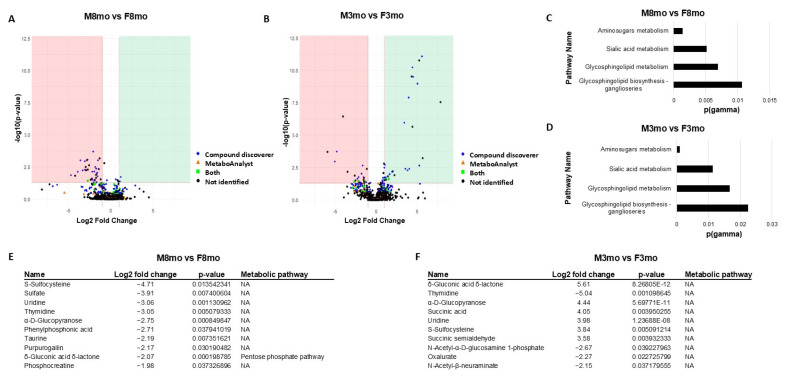
Metabolomics data comparing sex at two different ages through volcano plots, pathway enrichment analysis, and the top 10 statistically significant identified metabolites. (**A**) Sex comparison volcano plot of M8mo vs. F8mo; blue (compound discoverer matched), orange (MetaboAnalyst empirical compound matched), green (both the compound discoverer and MetaboAnalyst empirical compound matched). (**B**) Sex comparison volcano plot of M3mo vs. F3mo; blue (compound discoverer matched), orange (MetaboAnalyst empirical compound matched), green (both the compound discoverer and MetaboAnalyst empirical compound matched). (**C**) Eight-month-old sex comparison pathway enrichment analysis with statistically significant *p* (gamma) containing pathways with at least three statistically significant metabolites. (**D**) Three-month-old sex comparison pathway enrichment analysis with statistically significant *p* (gamma) containing pathways with at least three statistically significant metabolites. (**E**) Table of the top statistically identified metabolites in the M8mo vs. F8mo comparison, including Log2 fold change, *p*-value, and respective metabolic pathways. (**F**) Table of the top statistically identified metabolites in the M3mo vs. F3mo comparison, including Log2 fold change, *p*-value, and respective metabolic pathways.

Using IC-MS/MS in negative mode, 1137 unique *m*/*z* values were obtained from medaka bone tissue across all groups. The comparisons revealed 265 significant differently expressed metabolites between 8-month-old females (F8mo) vs. 3-month-old females (F3mo), 213 between 8-month-old males (M8mo) vs. 3-month-old males (M3mo), 36 between M8mo vs. F8mo, and 54 between M3mo vs. F3mo. Compound Discoverer positively identified 19.08% (217 compounds) of the *m*/*z* values. Additionally, MetaboAnalystR’s mummichog2 algorithm revealed that 3.43% (39 compounds) of the identified *m*/*z* values corresponded to the zebrafish library. Metabolites identified through both methods included O-Acetylserine, N-Glycolylneuraminic acid, Inosinic acid, dUDP, D-Sedoheptulose 7-phosphate, D-myo-Inositol 1,4-bisphosphate, Cytidine 5′-monophosphate-N-acetylneuraminic acid, Cytidine monophosphate, Adenosine monophosphate, 3-beta-D-galactosyl-sn-glycerol, and Glycerophosphoinositol. This overlap is also represented in volcano plots characterizing statistical differences among pairwise comparisons (Figure 2 and Figure 3).

### 3.2. Age-Related Metabolomic Changes

The age comparison for males revealed 141 upregulated *m*/*z* values and 72 downregulated *m*/*z* values in M8mo compared to M3mo, while for females, it showed 217 upregulated *m*/*z* values and 48 downregulated *m*/*z* values in F8mo compared to F3mo. A total of eight pathways were found to be statistically significant enrichment with age for both sexes: Aminosugars metabolism, Glycerophospholipid metabolism, Phosphatidylinositol phosphate metabolism, De novo fatty acid biosynthesis, Keratan sulfate biosynthesis, Keratan sulfate degradation, Pentose phosphate pathway, and Glycosphingolipid biosynthesis-ganglioseries (Figure 2C). Five of the eight statistically significant enriched pathways in response to age are glycosylation pathways, notably Glycosphingolipids (GSLs), Glycosylphosphatidylinositol anchors (GPI anchors), O-glycans, N-glycans and keratin sulfate metabolism in both sexes. In the M8mo vs. M3mo age comparison 2′-Deoxyguanosine 5′-diphosphate, Adenosine 5′-monophosphate, N-Acetylneuraminate, and GM3 were found potentially upregulated, and Adenosine 5′-diphosphate, and Adenosine 3′,5′-bisphosphate were downregulated metabolites in the Glycosphingolipid biosynthesis-ganglioseries, the Pentose phosphate pathway, and the de novo fatty acid biosynthesis (Figure 2D). In the F8mo vs. F3mo age comparison Adenosine 5′-diphosphate, Adenosine 3′,5′-bisphosphate and 2′-Deoxyguanosine 5′-diphosphate were potentially upregulated, while Galactosylglycerol, GM2, Adenosine 5′-monophosphate and 2′-Deoxyguanosine 5′-monophosphate were downregulated for these three metabolic pathways (Figure 2D).

### 3.3. Metabolomic Changes Related to Sex

Sex comparisons revealed 29 upregulated *m*/*z* values and 25 downregulated *m*/*z* values for M3mo vs. F3mo (Figure 3A). The 8-month volcano plot sex comparison revealed two upregulated *m*/*z* values and 34 downregulated *m*/*z* values (Figure 3B). Both sex comparisons revealed four statistically significant pathways: glycosphingolipid biosynthesis–ganglioseries, glycosphingolipid metabolism, sialic acid metabolism, and aminosugars metabolism (Figure 3C,D). In the M8mo vs. F8mo sex comparison, metabolites with a male bias were Adenosine 5′-diphosphate, Adenosine 3′,5′-bisphosphate, 2′-Deoxyguanosine 5′-diphosphate, and N-Acetyl-D-glucosamine 6-phosphate, N-Acetyl-D-mannosamine 6-phosphate, N-Acetylneuraminate and GM3 indicating differences in the Aminosugars metabolism, the Sialic acid metabolism, and the Glycosphingolipid biosynthesis (Figure 3E). Similarly, the M3mo vs. F3mo sex comparison revealed Adenosine 5′-diphosphate, Adenosine 3′,5′-bisphosphate and 2′-Deoxyguanosine 5′-diphosphate upregulation in the males, and N-Acetyl-alpha-D-glucosamine 1-phosphate, N-Acetyl-D-mannosamine 6-phosphate N-Acetylneuraminate, GM3 in the females for the same three pathways (Figure 3F).

### 3.4. Glycosylation Pathways

**Figure 4:**
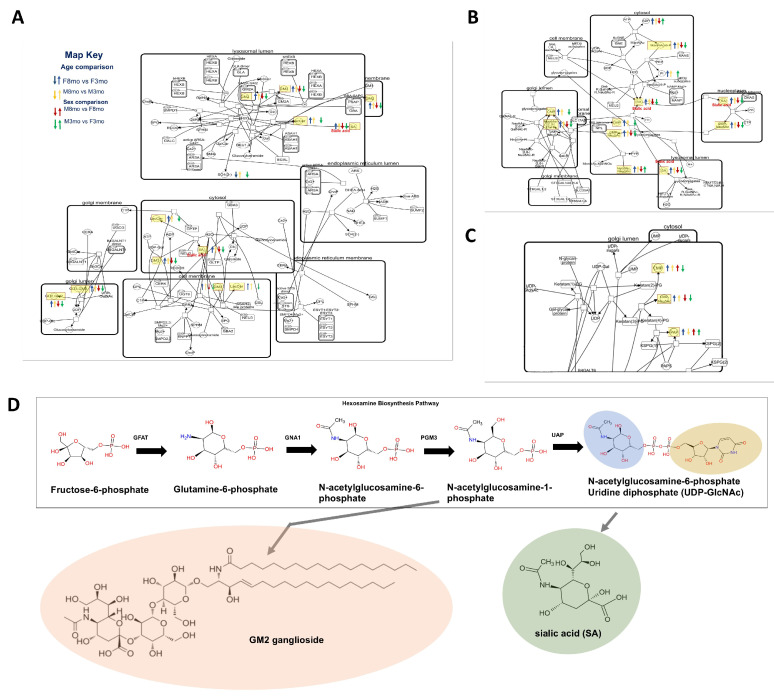
Pathway mapping of glycosphingolipids biosynthesis, sialic acid metabolism, and keratin sulfate biosynthesis across the four pairwise statistical comparisons. Metabolites were identified by MS/MS and peak list, and statistically significant deregulated metabolites are depicted by arrows (↑ = upregulated, ↓ = downregulated). The arrow color indicates the pairwise comparison: age (females = blue, males = yellow), sex (8mo = red, 3mo = green). (**A**) Visualization of glycosphingolipids biosynthesis. (**B**) Visualization of sialic acid metabolism. (**C**) Visualization of keratin sulfate biosynthesis. (**D**) The hexamine biosynthesis pathway from fructose-6-phosphate to a nucleotide-activated sugar (UDP-GlcNAc) is required in glycosylation pathways such as the synthesis of GM2 gangliosides and sialic acid. Non-ubiquitous metabolites are highlighted yellow and have a *p*< 0.05 and −1 < log2 fold change < 1.

Biological insights into pathway interconnectivity were gained by mapping pathways with statistically significant metabolites and their Log2 fold change. The observation of pathway regulation demonstrated interconnectivity between the glycosylation pathways, specifically between glycosphingolipids biosynthesis, sialic acid metabolism, and keratin sulfate biosynthesis (Figure 3). These glycosylation pathways were globally downregulated with respect to 8mo vs. 3mo and male vs. female bone tissue. The glycosylation pathways shared key metabolites such as sialic acid and UDP-GluNAc. This observation also extended to glycosphingolipid pathways (Figure S1, Supplementary Information), phosphatidylinositol phosphate metabolism (Figure S2, Supplementary Information), keratan sulfate biosynthesis (Figure S3, Supplementary Information), and glycoprotein biosynthesis within the O-glycan pathway (Figure S4, Supplementary Information). The central metabolites regulating the glycosylation network included Guanosine monophosphate (GMP) and sialic acid (Figure S5, Supplementary Information). Furthermore, age was shown to hyperglycosylate bone tissue through increased activity of the HPB, producing active sugars such as UDP-GlcNAc (Figure 4D). The excess presence of these active sugars is likely resulting in an abundance of metabolites like Ganglioside GM2 gangliosides and sialic acid in aging medaka (Figure 4A–C).

## 4. Discussion

Advancements in IC-high-resolution tandem MS have expanded the reach of untargeted metabolomics to include the important subgroup of complex sugars [20,31,35]. This metabolite subgroup has been largely overlooked in the skeletal tissue across sex and age. Only a few studies have investigated the global dysregulation of glycosylation metabolism driving tissue aging [24,36,37]. Here, we report evidence of hyperglycosylation occurring during bone tissue aging. The results demonstrate that hyperglycosylation is an age-associated phenotype, reinforcing the important role glycosylation pathways play in driving bone tissue aging in both females and males.

Glycolic modifications with age, while studied in osteoclasts and osteoblasts, are poorly understood throughout the entire bone tissue [2,22]. In aging males, additional enriched pathways were observed, including methylation, acetyl-CoA, nucleotides, and amino acid metabolism. Sex differences were less pronounced, with only four enriched pathways at 3mo and 8mo, three of which included glycosylation pathways. Overall, the baseline differences of metabolites in medaka bone tissue consisted largely of hyperglycosylation through glycosylation pathways, which was more pronounced with age. Specifically, increased activity in glycosylation pathways whose end byproducts are GPLs, GPIs, O-glycans, N-glycans, and keratin sulfate. The key metabolites driving this global glycosylation metabolism were UDP-sugars and sialic acid, which have the potential to facilitate regulation and metabolic crosstalk between HBP and glycosylation pathways, expanding our understanding of hyperglycosylation in skeletal tissue aging and health.

Bone aging, like that of other tissues, leads to inflammation and contributes to age-related bone loss [17,18]. A proteomic study has highlighted the significant role of immunology in this process, revealing that inflammation drives bone loss with age by inhibiting immune pathways such as IL-8 signaling and acute-phase response signaling [17]. The study’s ingenuity pathway analysis also noted disruptions in protein processing within the endoplasmic reticulum and changes in O-glycan biosynthesis. Despite these observations, the study did not provide a clear link or explanation regarding the glycosylation implications [17]. This gap resonates with our findings. Our research indicates that hyperglycosylation in bone tissue aging may interfere with normal cell-cell interactions and cellular signaling pathways, corroborating the proteomic study’s findings [17]. This interference is primarily driven by excessive glycolytic activity involving Glycosphingolipids (GSLs), Glycosylphosphatidylinositol (GPI), O-glycans, and N-glycans, all of which play crucial roles in fine-tuning inflammation and signaling pathways [17,38]. Changes in glycosylation have been associated with inflammation in the spine, spinal cord, and brain pathologies, as well as chronic inflammation [39,40]. On the other hand, reactive oxidative stress and tissue inflammation regulate enzymes, transporters, and chaperones involved in glycosylation [41,42,43,44,45]. Thus, further studies are needed to disentangle the temporal pattern of modulated glycosylation, cellular oxidative stress and occurrence of inflammation in aging tissues to identify the start ting point of the signaling cascade. The presented data in 8-month-old medakas show that glycosylation is modified most likely earlier or at the same time as inflamm-aging and sex-specific endocrine modifications are measured [1,23,24].

**Figure 5:**
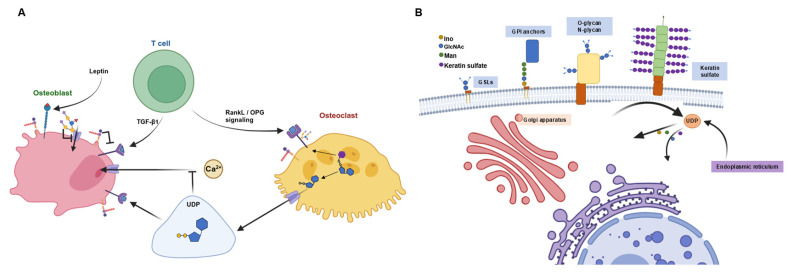
Schematic illustration of the extracellular and intracellular signaling role metabolism plays in bone tissue and cells. (**A**) The established extracellular role of metabolites signaling between osteocytes, osteoblasts, and osteoclasts. (**B**) Intracellular metabolism of glycosylation from the uptake of UDP and sugar monomers by the Golgi apparatus and endoplasmic reticulum for the biosynthesis of GSLs, GPI anchors, O-glycans, N-glycans, and keratin sulfate.

Recent studies have shown that bone signaling pathways, such as RANKL and Siglec-15, depend on this glycolytic fine-tuning through both post-transcriptional glycosylation and intermolecular interactions between GSLs and receptor/signaling proteins (Figure 5A) [1,23,24]. Consequently, the impaired signaling between osteoclasts, osteoblasts, and immune pathways in bone tissue with age can be linked to hyperglycosylation, highlighting a critical area for further investigation.

Intracellular hyperglycosylation can be attributed to dysfunction in the HBP, impacting the endoplasmic reticulum and Golgi apparatus (Figure 5B). Although our methods do not directly detect cell stress, a link can be inferred between glycolytic metabolic flux and endoplasmic reticulum stress. Unlike gene transcription or protein translation, glycosylation is not template-driven. It relies on the availability of nucleotide sugar precursors and glycosyltransferase enzymes [28,38]. Our research found a globally increased conversion of sugar precursors into nucleotide sugar precursors via HBP pathways in bone tissue from older animals, establishing UDP-sugars as a key metabolite in bone tissue aging. It has been shown that activation of HBP increases global O-GlcNAC acylation during RANKL-mediated osteoclast differentiation by interfering with NF-κB p65 and the Nuclear Factor of Activated T-cells [21]. Our findings further support the suggestion that O-GlcNAc acylation is extremely nutrient-sensitive because it requires UDP-GlcNAc, the product of the HBP [21]. We found that UDP-GlcNAc influence may also play a role at the intersection between cytoplasmic sugar activation and their transport to the endoplasmic reticulum and Golgi apparatus (Figure 5B). Senescent drove metabolic flux, leading to an increase of UDP-sugars feeding into glycosylation pathways, leading to the excessive production of glycosphingolipids and sialic acid in older individuals regardless of sex. This aging effect may also amplify bone loss by UDP’s role in extracellular signaling between osteoclasts and osteoblasts through the P2RY14 purinergic receptor (P2Y14) (Figure 5A) [40]. The binding of UDP to P2Y14 has been shown to decrease Ca2+ in response to mechanical stress, contributing to bone loss [27]. Downstream of the HBP pathway is sialic acid, another pivotal metabolite for explaining the observed senescence hyperglycosylation phenotype. Mechanistically, all the cell–cell interactions and cellular signaling pathways discussed rely on the decoration of macromolecules with sialic acid and include the glycosylation products GSLs, GPI, O-glycans, and N-glycans. Deregulation of the sialic acid metabolism has been associated with inflammation [46]. Our data further support the application of sialic acid upregulation as a marker for age-related inflammation in bone cells [46]. Moreover, the data hint towards the use of sialylation inhibitors as a glycotherapeutic measure to reduce age-induced bone inflammation [46,47]. Further research is needed to elucidate and explore the complex biomolecule products at the ends of the GSL, GPI, O-glycan, and N-glycan pathways, which are increased as a result of the SASP hyperglycosylation phenotype.

## 5. Conclusions

Unraveling the role of hyperglycosylation in bone tissue aging not only provides mechanistic insights into inflammation and bone loss but also translates into several medical applications. This knowledge opens potential avenues for the development of novel glycan-based biomarkers, such as UDP-sugars and sialic acid, for the identification of bone aging.

## Data Availability

The raw data and codes are available under Rlabeille/Bone-Metabolomics-Medaka-Sex-and-Age-study (github.com) (accessed on 22 September 2024).

## Supplementary Materials

The following supporting information can be downloaded at https://www.mdpi.com/article/10.3390/metabo14100525/s1, Figure S1: SBGN pathways mapping of Glycosphingolipid biosynthesis for the sex and age comparison; Figure S2: SBGN pathways mapping of Phosphatidylinositol phosphate metabolism for the sex and age comparison; Figure S3: SBGN pathways mapping of Keratan sulfate biosynthesis for the sex and age comparison; Figure S4: SBGN pathways mapping of O-Glycan biosynthesis for the sex and age comparison; Figure S5: SBGN pathways mapping of Sialic acid metabolism for the sex and age comparison. GitHub for code and data https://github.com/Rlabeille/Bone-Metabolomics-Medaka-Sex-and-Age-study (accessed on 22 September 2024).
