# Supplementary material for: Hyperglycosylation as an Indicator of Aging in the Bone Metabolome of Oryzias latipes
Source: Metabolites. 2024 Sep 27;14(10):525. doi: 10.3390/metabo14100525 (PMC11509322; doi:10.3390/metabo14100525)
Supplement: Supplementary file 1 [file metabolites-14-00525-s001.zip › metabolites-3200350-supplementary.pdf]

Supplementary information: Hyperglycosylation as an indicator of aging in the bone metabolome of Oryzias latipes

Remi Labeille[1], Justin Elliot[1], Hussain Abdulla[1], Frauke Seemann[2,*]

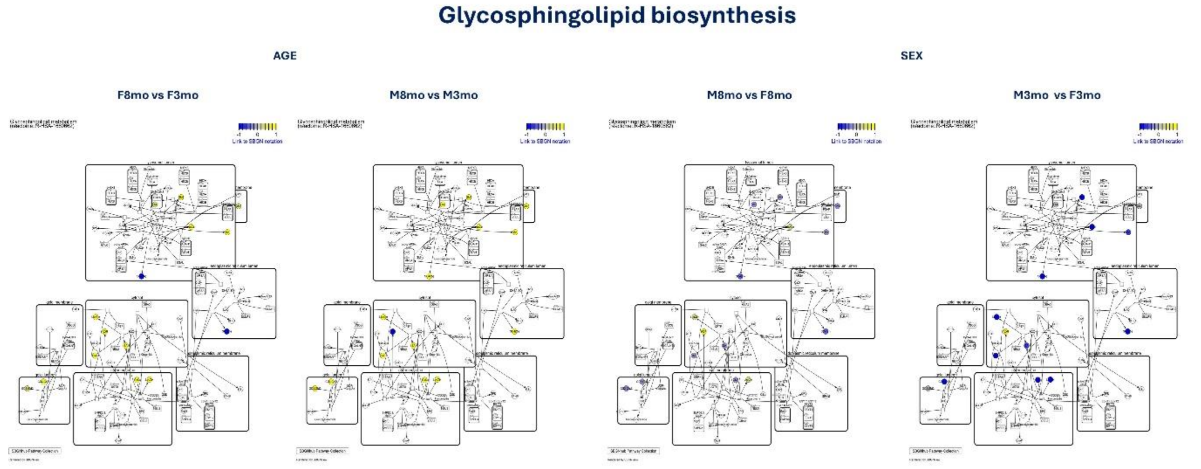

Figure S1. SBGN pathways mapping of Glycosphingolipid biosynthesis for the sex and age comparison. Metabolite hits with a p-value less than or equal to 0.05 from both compound discoverer MS/MS identification and MetaboAnalystR peak list identification shows on a spectrum from blue = -1log2 fold change to yellow = 1log2 fold change.

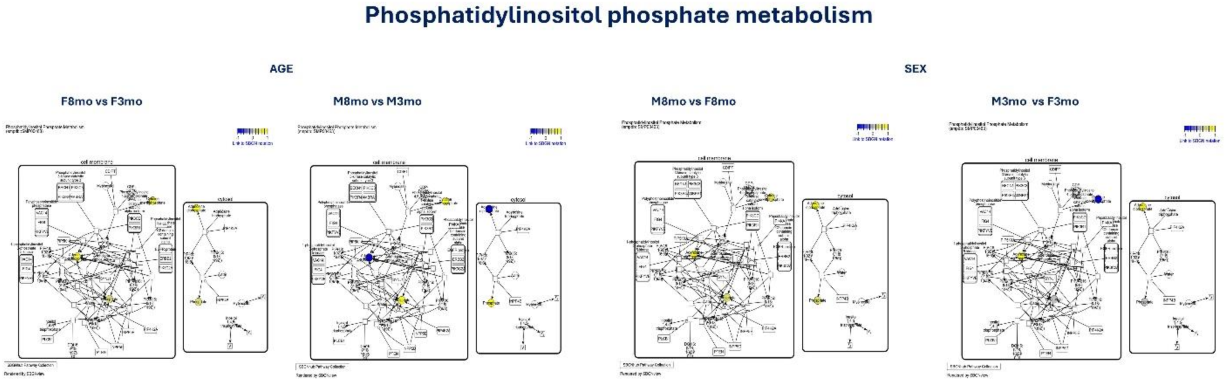

Figure S2. SBGN pathways mapping of Phosphatidylinositol phosphate metabolism for the sex and age comparison. Metabolite hits with a p-value less than or equal to 0.05 from both compound discoverer MS/MS identification and MetaboAnalystR peak list identification shows on a spectrum from blue = -1log2 fold change to yellow = 1log2 fold change.

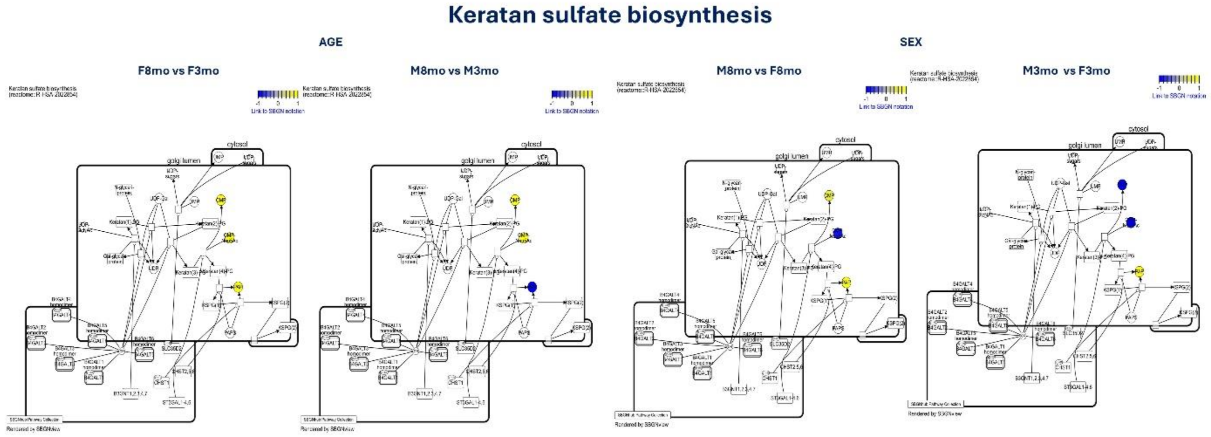

Figure S3. SBGN pathways mapping of Keratan sulfate biosynthesis for the sex and age comparison. Metabolite hits with a p-value less than or equal to 0.05 from both compound discoverer MS/MS identification and MetaboAnalystR peak list identification shows on a spectrum from blue = -1log2 fold change to yellow = 1log2 fold change.

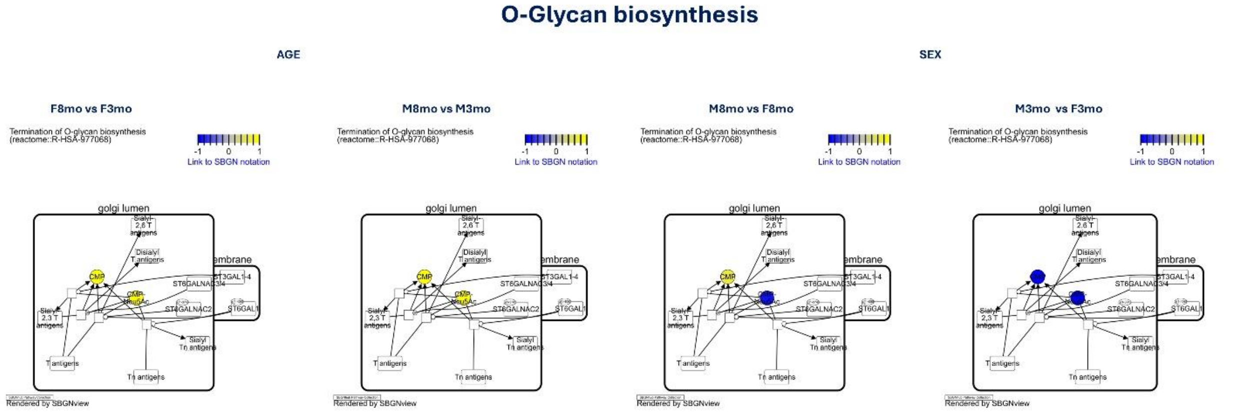

Figure S4. SBGN pathways mapping of O-Glycan biosynthesis for the sex and age comparison. Metabolite hits with a p-value less than or equal to 0.05 from both compound discoverer MS/MS identification and MetaboAnalystR peak list identification shows on a spectrum from blue = -1log2 fold change to yellow = 1log2 fold change.

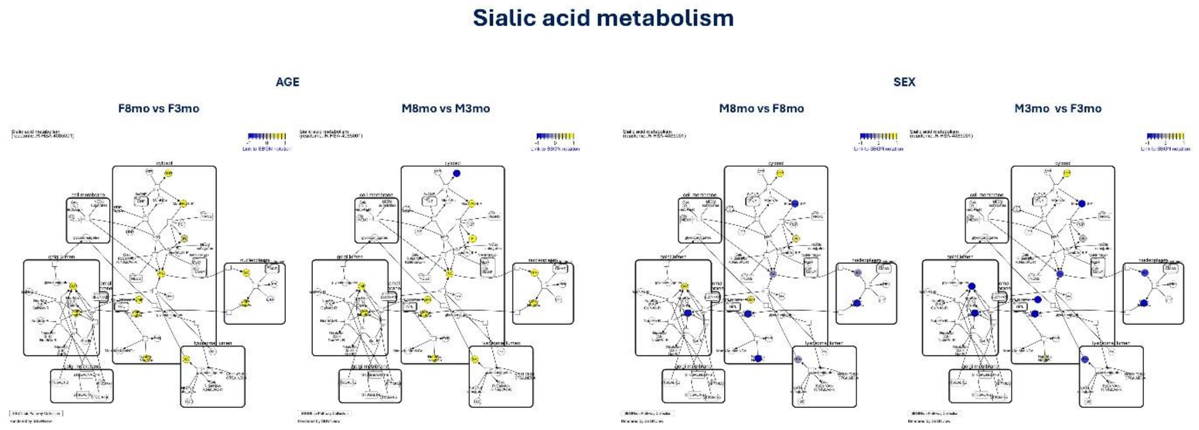

Figure S5. SBGN pathways mapping of Sialic acid metabolism for the sex and age comparison. Metabolite hits with a p-value less than or equal to 0.05 from both compound discoverer MS/MS identification and MetaboAnalystR peak list identification shows on a spectrum from blue = -1log2 fold change to yellow = 1log2 fold change.

.
